# The *Cdh5-CreERT2* transgene causes conditional *Shb* gene deletion in hematopoietic cells with consequences for immune cell responses to tumors

**DOI:** 10.1038/s41598-019-44039-z

**Published:** 2019-05-17

**Authors:** Qi He, Xiujuan Li, Kailash Singh, Zhengkang Luo, Mariela Meija-Cordova, Maria Jamalpour, Björn Lindahl, Vitezslav Kriz, Reetta Vuolteenaho, Maria Ulvmar, Michael Welsh

**Affiliations:** 1 Department of Medical Cell Biology, Uppsala University, Uppsala, Sweden; 2 Cyrus Tang Hematology Center, Soochow University, Suzhou, China; 3 Institute of Molecular Genetics of the CAS, Prague, Czech Republic; 4 Transgenic Animals Facility, Oulu University, Oulu, Finland; 5 Department of Immunology, Genetics and Pathology, Uppsala University, Uppsala, Sweden

**Keywords:** Animal disease models, Cancer models

## Abstract

The tamoxifen-responsive conditional *Cdh5-CreERT2* is commonly used for endothelial cell specific conditional deletion of loxP-flanked gene sequences. To address the role of endothelial cell *Shb* gene for B16F10 melanoma immune responses, tamoxifen-injected *Cdh5-CreERT2*/WT and *Cdh5-CreERT2*/*Shb*^*flox/flox*^ mice received subcutaneous tumor cell injections. We observed a decrease of tumor myeloid cell *Shb* mRNA in the tamoxifen treated *Cdh5-CreERT2*/*Shb*^*flox/flox*^ mice, which was not present when the mice had undergone a preceding bone marrow transplantation using wild type bone marrow. Differences in CD4+/FoxP3+ Tregs were similarly abolished by a preceding bone marrow transplantation. In *ROSA26-mTmG* mice, *Cdh5-CreERT2* caused detectable floxing in certain bone marrow populations and in spleen cells. Floxing in bone marrow could be detected two months after tamoxifen treatment. In the spleen, however, floxing was undetectable two months after tamoxifen treatment, suggesting that *Cdh5-CreERT2* is operating in a non-renewable population of hematopoietic cells in this organ. These data suggest that conditional gene deletion in hematopoietic cells is a potential confounder in experiments attempting to assess the role of endothelial specific effects. A cautious approach to achieve an endothelial-specific phenotype would be to adopt a strategy that includes a preceding bone marrow transplantation.

## Introduction

The *Shb* gene^1^ plays a role in tumor biology in numerous settings^2–8^. Many of the findings point to an endothelial cell involvement^2,3,9,10^, but the Src-homology 2 domain protein B (SHB) also has an impact on immune or hematopoietic cell behaviour^11–14^. *Shb* is required for vascular endothelial growth factor-A (VEGFA) dependent angiogenesis and vascular leakage in endothelial cells^9^ and these effects appear to be mediated via regulation of focal adhesion kinase^2,15^. T-cell receptor activation^12^ also requires *Shb* and in the absence of *Shb*, T cells exhibit an augmented Th2-response^14^. Absence of *Shb* decreases hematopoietic stem cell proliferation causing a reduced ability of myeloid cells to repopulate after bone marrow replacement^13^. Our recent finding that CD8+ cell infiltration into B16F10 melanomas was influenced by the *Shb* gene raised the possibility that endothelial cells exert an influence on immune responses to tumors in a manner that could be of relevance to tumor expansion and metastasis^4^, and we decided to investigate this further by crossing the *Cdh5-CreERT2(1Rha)* transgene Cre-recombinase onto the *Shb*^*flox/flox*^ background. This transgene is considered the gold standard for endothelial specific conditional deletion of loxP target genes^16^ and has not been reported to generate inactivation of hematopoietic cells in adult mice unlike the Tie2-Cre transgene which efficiently causes gene deletion in hematopoietic cells^17^ or the constitutive *Cdh5-Cre* transgene that causes recombination in embryonic hematopoietic cells^18^. One report suggests *Cdh5-Cre*-dependent recombination in hematopoietic cells in adult mice but that study used a different *Cdh5* promoter fragment to drive Cre expression^19^. A third transgenic *Cdh5-CreERT2* mouse was generated that perturbed angiogenesis but was not further investigated in detail with respect to its capacity to cause non-endothelial cell gene inactivation^20^. Herein we observed that the *Cdh5-CreERT2* transgene^16^ causes conditional gene deletion in certain hematopoietic cells with functional consequences that can be avoided by implementing protocols utilizing a preceding bone marrow transplantation.

## Results and Discussion

**Figure 1:**
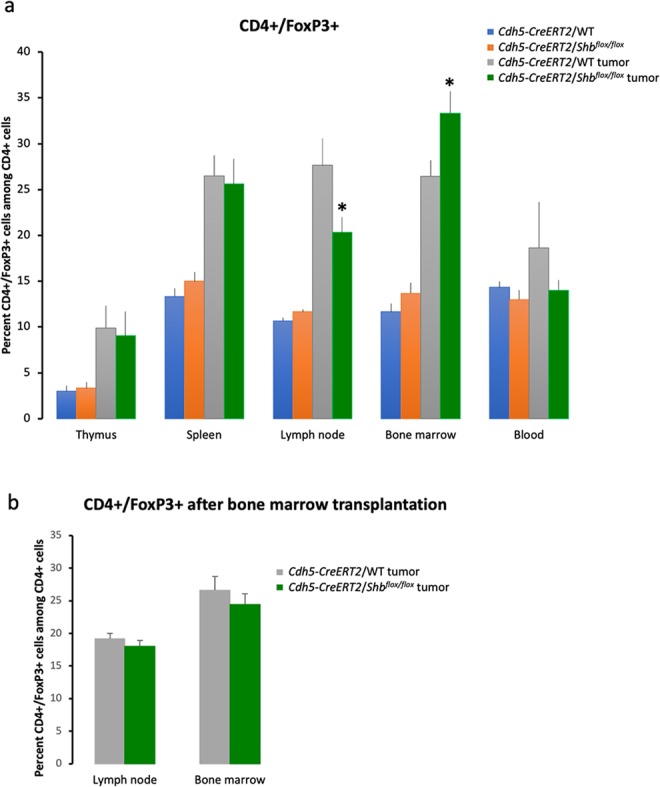
Expression of CD4+/FoxP3+ Tregs in different immune organs in response to B16F10 melanoma growth. (**a**) Conditionally deleted *Shb* and corresponding wild type controls with or without tumors were sacrificed and immune organs collected. The percentages CD4+/FoxP3+ cells were determined in percent of parental populations (which did not differ between the experimental conditions). Means ± SEM are given for n = 4 (non-tumor) or n = 10 (tumor) of each genotype in three separate experiments. The experimental groups of each organ were subjected to one-way ANOVA to reject the null hypothesis (p < 0.01 for lymph node and p < 0.001 bone marrow) followed by Sidak’s multiple comparisons test to compare WT tumor with conditionally deleted *Shb* tumor. *Indicates p < 0.05 when compared with corresponding tumor wild type control. (**b**) CD4+/FoxP3+ cells in lymph nodes and bone marrow of tumor bearing mice that had received a wild type bone marrow transplantation 3 months prior to the tumor experiment. Means ± SEM for n = 5 of each genotype are given in one experiment. P values were 0.44 and 0.36, respectively.

**Figure 2:**
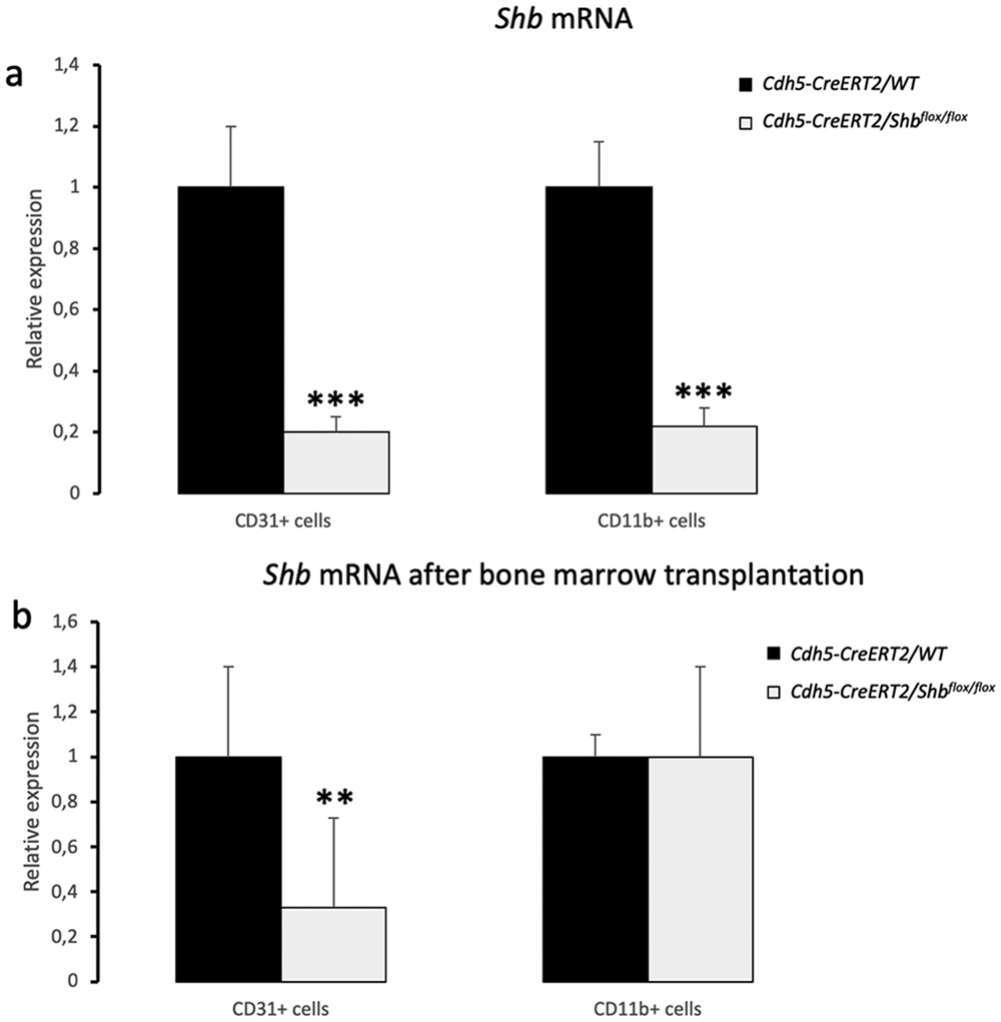
*Shb* mRNA in tumor CD31+ and CD11b+ cells from *Cdh5-CreERT2/WT and Cdh5-CreERT2/Shb*^*flox/flox*^ mice. (**a**) *Shb* mRNA with or without conditional deletion of the *Shb* gene by qPCR. Relative values compared with corresponding wild type controls are given as means ± SEM for n = 10 in three separate experiments. ***Indicates p < 0.001 by Students’ t-test when compared with corresponding wild type control. (**b**) *Shb* mRNA after bone marrow transplantation. N = 5 of each genotype in one experiment and **indicates p < 0.01 when compared with wild type control by a Students’ t-test.

Mice (*Cdh5-CreERT2*/WT or *Cdh5-CreERT2*/*Shb*^*flox/flox*^ pretreated with tamoxifen) with B16F10 melanomas were investigated for endothelial-dependent alterations in Treg immune cells as a consequence of *Shb* gene inactivation. Immune organs (thymus, inguinal lymph nodes, spleen, bone marrow and blood) were collected and subjected to immune profiling by FACS staining to detect CD4/FoxP3 double-positive Tregs. Absence of *Cdh5-CreERT2* induced deletion of *Shb* in endothelial cells reduced a tumor-induced increase of CD4+/FoxP3+ Tregs in local lymph nodes and accentuated that cell population in bone marrow (Fig. 1a). Next, these analyses were supplemented with bone marrow transplantation experiments using wild type bone marrow to *Cdh5-CreERT2*/*Shb*^*flox/flox*^ or *Cdh5-CreERT2*/WT mice. The shift in the CD4+/FoxP3+ population caused by absence of *Shb* disappeared (Fig. 1b) suggesting that the effects were cell autonomous to Tregs. Isolated endothelial (CD31+) cells from tumors showed a 75% reduction of *Shb* mRNA by qPCR (Fig. 2a). Unexpectedly, an identical reduction of *Shb* mRNA in CD11b+ cells was noted (Fig. 2a) and such a reduction was observed regardless of whether compared with wild type mice (Fig. 2a) or *Shb*^*flox/flox*^ mice (S. Fig. 1A). The cell populations were highly enriched for VE-cadherin/*Cdh5* and CD11b (*Itgam*) (S. Fig. 1B,C), respectively, excluding endothelial cell contamination as an explanation for the reduction in *Shb* mRNA in CD11b+ cells. The reduction in tumor CD11b+ *Shb* mRNA was also reverted by wild type bone marrow (Fig. 2b). This suggests conditional deletion of *Shb* in hematopoietic cells by the *Cdh5-CreERT2* transgene as responsible for the effects.

We next investigated floxing of *Shb*^*flox/flox*^ in bone marrow CD31+ and CD11b+ cells by genotyping and detected gene inactivation in both cell types at day 4 after the cessation of the tamoxifen injections in adult mice (S. Fig. 2). Three single-cell RNAseq studies on hematopoietic bone marrow cells in adult mice have detected expression of VE-cadherin/*Cdh5* mRNA, albeit at a low level/frequency^21–23^ and the following resources of single cell RNAseq in B16F10 tumors and mouse bone marrow have similarly demonstrated low Cdh5 expression in sparse non-endothelial cells (https://www.ebi.ac.uk/gxa/sc/experiments/E-EHCA-2/Results?geneId=ENSMUSG00000031871&k=16&clusterId=%5B1%5D; https://www.ebi.ac.uk/gxa/sc/experiments/E-GEOD-100426/Results?geneId=ENSMUSG00000031871).

**Figure 3:**
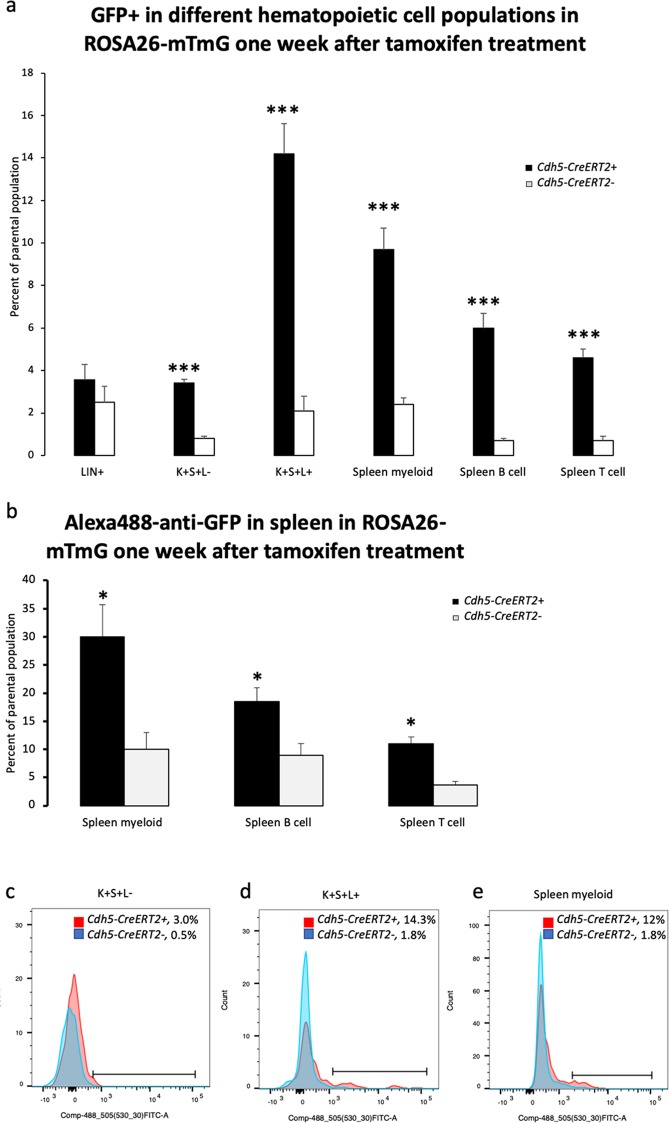
(**a**) GFP+ percentage in different bone marrow and spleen hematopoietic cell populations. *ROSA26-mTmG* mice with or without the *Cdh5-CreERT2* transgene were pre-treated with tamoxifen and maintained for seven days before sacrifice. Means ± SEM are given. Values in mice without *Cdh5-CreERT2* reflect autofluorescence. ***Indicates p < 0.001 when compared with corresponding control without the *Cdh5-CreERT2* transgene by a Students’ t-test. LIN+ = lineage positive bone marrow cells; K + S + L− = cKit positive, Sca1 positive and lineage negative (LSK) bone marrow cells; K + S + L+ = cKit positive, Sca1 positive and lineage positive bone marrow cells; Spleen myeloid = Gr1 positive/CD11b positive spleen cells; Spleen B cell = B220 positive/CD19 positive spleen cells; Spleen T cell = CD4 positive/CD8 positive spleen cells. N = 4 for the bone marrow and n = 5 for the spleen populations in three separate experiments. (**b**) Staining spleen cells for GFP using an anti-GFP-FITC antibody. After staining of spleen cells for lineage markers as in Fig. 3a, cells were stained for GFP prior to FACS. Values as percentage of parental cell population are means ± SEM. *Indicates p < 0.05 when comparing *Cdh5-CreERT2* mice with wild type controls without *Cdh5-CreERT2* (autofluorescence) by a Students’ t-test. N = 5 in three separate experiments. (**c**) Histogram showing *Cdh5-CreERT2*+ (red) and *Cdh5-CreERT2-* (blue) GFP + K + S + L− cells with the corresponding gated values given. (**d**) Histogram showing *Cdh5-CreERT2*+ (red) and *Cdh5-CreERT2-* (blue) GFP + K + S + L+ cells with the corresponding gated values given. (**e**) Histogram showing *Cdh5-CreERT2*+ (red) and *Cdh5-CreERT2-* (blue) GFP+ spleen myeloid cells with the corresponding gated values given.

**Figure 4:**
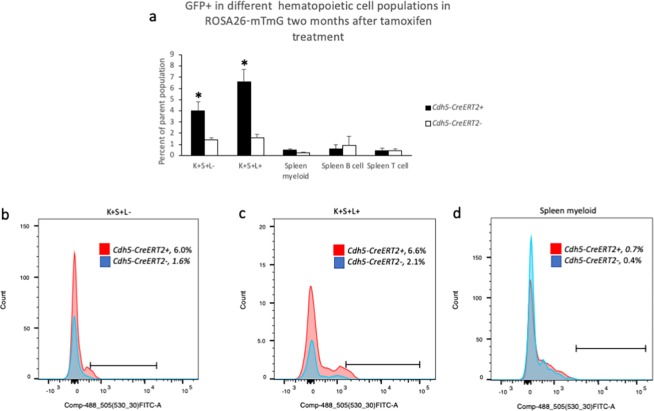
(**a**) GFP+ cells in different hematopoietic cell populations as in Fig. 3 two months after tamoxifen treatment. *ROSA26-mTmG* mice with or without the *Cdh5-CreERT2* transgene were pre-treated with tamoxifen and maintained for two months before sacrifice. Means ± SEM for *Cdh5-CreERT2+* (n = 5) and *Cdh5-CreERT2-* (n = 3) in two separate experiments are given. Values in mice without *Cdh5-CreERT2* reflect autofluorescence. *Indicated p < 0.05 compared with corresponding *Cdh5-CreERT2-* values by Students’ t-test. K + S + L− = cKit positive, Sca1 positive and lineage negative (LSK) bone marrow cells; K + S + L+ = cKit positive, Sca1 positive and lineage positive bone marrow cells; Spleen myeloid = Gr1 positive/CD11b positive spleen cells; Spleen B cell = B220 positive/CD19 positive spleen cells; Spleen T cell = CD4 positive/CD8 positive spleen cells. (**b**) Histogram showing *Cdh5-CreERT2*+ (red) and *Cdh5-CreERT2-* (blue) GFP + K + S + L− cells with the corresponding gated values given. (**c**) Histogram showing *Cdh5-CreERT2*+ (red) and *Cdh5-CreERT2-* (blue) GFP + K + S + L+ cells with the corresponding gated values given. (**d**) Histogram showing *Cdh5-CreERT2*+ (red) and *Cdh5-CreERT2-* (blue) GFP+ spleen myeloid cells with the corresponding gated values given. The corresponding GFP/tdTomato plots to (**b**–**d**) in wild type mice without ROSA26-mTmG are shown in Supplementary Figs 8–10.

To directly demonstrate *Cdh5-CreERT2* activity in hematopoietic cells, the *ROSA26-mTmG* reporter mouse was used together with the transgene. This reporter expresses under basal conditions tdTomato and begins expressing GFP after floxing. However, the tdTomato signal is stable and may remain for weeks after Cre recombination^24^. The GFP/FITC signal was intermediate (10^3^–10^4^) in bone marrow cells (S. Figs 3, 4) and consequently there was no distinct difference between GFP-positive cells and autofluorescence. For that reason, Cre-negative controls were always used in parallel under identical conditions. In the bone marrow, lineage-positive cells lacked recombination that showed statistical significance seven days after tamoxifen injections (Fig. 3). In cKit + Sca1 + Lineage− (K + S + L−) stem and multi-potent progenitor cells a low but detectable degree of recombination occurred that was consistently higher than that of the *Cdh5-CreERT2* negative cells using the identical gating for GFP+ (Fig. 3, S. Fig. 3). Another bone marrow population with detectable recombination was a hitherto uncharacterized cKit + Sca1 + Lineage+ cells (Fig. 3, S. Fig. 4). We could not detect significant endothelial cell contamination in the K + S + L− population by FACS analysis (S. Fig. 3) since there were no or very few GFP^hi^-cells (>10^4^) present in that population, which is in contrast to what was observed in lung endothelial cells in which the GFP/FITC FACS-signal is >10^4^ (S. Fig. 5) when analyzed under identical conditions. The low degree of endothelial cell contamination could be a consequence of bone marrow crushing without digestion during preparation and the gating for high cKit expression. Standard protocols for bone marrow endothelial cell isolation include enzymatic digestion^25,26^ and endothelial cell cKit expression is lower than that of cKit + Sca1 + Lineage− hematopoietic stem/multipotent progenitor cells^22,27^.

In spleen, myeloid cells (CD11b+/Gr1+), B cells (CD19+/B220+) and T cells (CD4+/CD8+) all exhibited recombination of the *ROSA26-mTmG* locus and the effect became more apparent by fixing the cells and staining for GFP (Fig. 3, S. Fig. 6). The expression of the *ROSA26-mTmG* reporter was significantly lower in hematopoietic (CD45+/CD31+) cells than in endothelial cell when flow cytometry was performed under identical conditions in the same FACS analysis (S. Fig. 5, results not shown). Lung CD45/CD31-positive hematopoietic cells also showed detectable recombination (S. Fig. 5) comparable to that of spleen myeloid cells (S. Fig. 6). When ROSA26-mTmG recombination was analyzed two months after tamoxifen treatment, floxing was detected in the bone marrow populations, whereas floxing could not be detected in the spleen (Fig. 4). This suggests that floxing in spleen occurs in a non-renewable hematopoietic population in the spleen.

In summary, the *Cdh5-CreERT2* transgene results in conditional deletion in hematopoietic cells. The effect may seem modest as suggested by the *ROSA26-mTmG* reporter data ranging between 3–20% but could play a significant role in certain settings. The data are based on two separate *Cdh5-CreERT2* sublines excluding the possibility that unique drifting in phenotypic properties has occurred during local breeding. Firstly, differences between different hematopoietic cell types are noted. A partial explanation may lie in differences in *Cdh5-CreERT2* expression but also in how susceptible the locus is to recombination in different cells^28,29^. In addition, different loci may show different susceptibility to floxing^29^. Another issue is whether there is positive or negative selection for the hematopoietic cell with a gene inactivation causing altered cellular properties. Finally, the pathological condition at which the study is conducted may influence the efficacy of conditional deletion. Currently in tumor CD11b+ cells, 75% of these appear to have deleted their *Shb* gene based on mRNA content and this had phenotypic consequences since tumor-induced alterations in Tregs were observed.

There are several reasons why *Cdh5-CreERT2*^16^ activity in hematopoietic cells has remained hitherto undetected. The tdTomato signal is very stable so one must gate tdTomato/GFP double positive cells and due to the relatively weak GFP signal these are only moderately above background fluorescence as depicted by the Cre-negative cells. In addition, the population most active in the bone marrow is an uncharacterized lineage+/Sca1+/cKit+ population probably reflecting a progenitor subtype. Finally, conditional deletion is inititally (after one week) readily detected in spleen and undetectable two months later.

The potential of conditional deletion in hematopoietic cells can easily be avoided by performing a preceding bone marrow transplantation but that has potential adverse effects as well. Besides the simple fact that the mice will be of older age since complete hematopoietic restitution takes three months and causes changes in the relative proportions of different hematopoietic subpopulations^30^, the irradiation has direct adverse effects on endothelial cells^31^.

## Conclusion

The *Cdh5-CreERT2* transgene may conditionally delete target genes in certain hematopoietic cells and in order to avoid possibly confounding results, a strategy employing a preceding bone marrow transplantation can be implemented.

## Materials and Methods

### Mice

The conditional *Shb*^*flox*^ (Shb^tm1a(EUCOMM)Hmgu^) mouse on the C57Bl/6 strain was generated by the Intrafrontier I3 mouse project at the Biocenter Transgenic Core Facility, University of Oulu, Oulu, Finland. The C57BL/6N embryonic stem cell clone HEPD0613_4_G08-1 targeted with the “knock-in-first” construct was obtained from EUCOMM, see (http://www.mousephenotype.org/data/genes/MGI:98294)^32^. The tm1a allele contains an IRES:*lacZ* trapping cassette and a floxed promoter-driven *neo* cassette inserted into an intron of the *Shb* gene. The clone was characterized by chromosome, copy number and internal PCR analysis (http://www.mousephenotype.org/data/genes/MGI:98294) and Southern blot analysis. The ES cell clone HEPD0613_4_G08-1 was injected into laser-treated morulas (Zyrcos, Hamilton Thorne) that were transferred into pseudopregnant recipients. The chimeric mice were backcrossed with C57BL/6 mice, initially with C57Bl/6OlaHsd and after arrival to Uppsala heterozygous mice were bred with C57Bl/6J for four generations. The conditional construct sequence can be found at https://www.ncbi.nlm.nih.gov/nuccore/JN955283.1?report=genbank&to=37884 and the map of the vector at https://www.i-dcc.org/imits/targ_rep/alleles/19006/vector-image.

The *Cdh5-CreERT2* transgenic mouse was kindly provided by Dr Ralf Adams, Max Planck Institute for Molecular Biomedicine, Münster, Germany and imported on two separate occasions to Uppsala University^16^. One of the imports was used for the conditional deletion of *Shb* (*Cdh5-CreERT2/Shb*^*flox*^ (Shb^tm1b(EUCOMM)Hmgu^) and the other for crossing with *ROSA26-mTmG* (B6.129(Cg)-Gt(ROSA)26Sor^tm4(ACTB-tdTomato,-EGFP)luo^)^33^ (https://www.jax.org/strain/007676) to assess Cre recombination. All mice used for experimentation in this study were older than 8 weeks.

All animal experiments were approved by the Regional State Administrative Agency for Southern Finland and by the local animal ethics committee at the Uppsala County Court. EU regulations and guidelines for housing and care of laboratory animals were followed (DIRECTIVE 2010/63/EU on the protection of animals used for scientific purposes).

### Genotyping

The primers used for genotyping were: ShbF2: 5′ GGC ACT TCT GGA GAG CAG TAG A 3′; ShbR3: 5′ ACA GTG TGA GGC GAT CTC TGG 3′; Construct primer: 5′ TCG TGG TAT CGT TAT GCG CC 3′ with annealing at 60 °C. The mutant generates a band of 436 bp whereas the wild type a band of 741 bp. For demonstrating conditional deletion, the primers used were 5′-AAT AAT AAC CGG GCA GGG GG and 5′-GGC ATG GCT TGA ATG TGC TC at an annealing temperature of 65 °C amplifying a 498 base pair product upon amplification of the floxed gene. This PCR reaction does not detect non-floxed *Shb*^*flox/flox*^ due to the large size of the anticipated product (>3 kb).

### Bone marrow transplantation

Bone marrow cells were collected from 8–10 weeks old wild type donor mice and transplanted to lethally irradiated (two times 4.5 Gy two hours apart) recipients (10 weeks old) of *Cdh5-CreERT2/WT* (wild type) or *Cdh5-CreERT2*/*Shb*^*flox/flox*^ (5 mice each) as described^4^. The mice were maintained for 3 months before further experimentation.

### Tumor studies

B16F10 melanoma cells (2 × 10^5^) were injected subcutaneously as described^4^ in mice of 8–12 weeks of age that were injected for five consecutive days with 2 mg tamoxifen dissolved in peanut oil ten days after the last tamoxifen injection. Alternatively, mice subjected to a bone marrow transplantation three months prior with wild type bone marrow were used. When tumors reached a size of 0.5–1 cm^3^ mice were sacrificed and relevant organs collected.

### Immune cell profiling

Thymi, spleens, inguinal lymph nodes, bone marrows and blood were collected and cells isolated that were stained for CD4 and FoxP3 and analyzed by fluorescence activated cell sorting (FACS) as described^5^.

### Cell isolation by magnetic beads

Tumors were excised and digested as described^4^. Alternatively, iliac and femur bones were crushed and cells were collected by washing with PBS. Spleens were disrupted with a 1 ml syringe and cells collected in PBS. Blood was drawn immediately before sacrifice. Bone marrow, spleen and blood was subjected to red blood cell lysis by incubation for 15 minutes on ice in red blood cell lysis buffer (150 mM NH_4_Cl, 10 mM NaHCO_3_, 1 mM EDTA). Collected non-lysed cells (10^7^) in 180 μl were incubated with 10 μl CD11b microbeads (130-049-601, MACS Miltenyi Biotec, Germany) for 15 minutes at 4 °C. After washing the cells were applied to a MS column (130-042-201, MACS Miltenyi), washed again and eluted. The original flow-through upon column application was collected, incubated with 4 μl biotin-anti-CD31 (102504, Biolegend) plus 20 μl anti-biotin microbeads (130-090-485, MACS Miltenyi) and further column purified as above. RNA from eluted cells was isolated using the RNeasy mini-kit (Qiagen, Hilden, Germany). Lung endothelial cells were isolated as described^15^ by collagenase digestion and purification on CD31+ beads.

### Real-time reverse transcriptase PCR (qPCR)

Gene expression was determined using the QuantiTect^TM^ SYBRGreen real-time RT-PCR kit (Qiagen) as described^4,5^. For *Shb*, 5′-TTT GAT GCC AAG AGC GAC CT and 5′-GAG TCT GAG TCC ACG CTC TG primers were used. For bone marrow VE-cadherin/*Cdh5* mRNA, the following primer pairs were used: 5′-TTG CCC TGA AGA ACG AGG AC; 5′-ACT GCC CAT ACT TGA CCG TG and 5′-AGC AGT GGA TGC AGA TGA CC; 5′-GCC TGT TTC TCT CGG TCC AA. Beta-actin values were subtracted and relative expression was determined according the formula 2^−∆Ct^ compared with relevant control.

### Flow cytometry

The transgenic mice were injected for five consecutive days with tamoxifen (2 mg/mouse) and maintained for seven days before sacrifice. Bone marrow cells were stained with purified antibodies recognizing lineage markers (CD3, CD4, CD8, Gr1, CD11b, B220, CD19 and Ter119) followed by an anti-rat secondary antibody (PerCP-Cy5.5, Biolegend). Subsequently, cells were stained for cKit (APC-Cy7, eBioscience) and Sca1 (APC, eBioscience). Spleen and blood cells were stained for lineage (Gr1 and CD11b; B220 and CD19; CD4 and CD8) followed by anti-rat secondary antibody. Stained cells were subjected to flow cytometry by FACS essentially as described^13^. Spleen cells were fixated in 4% paraformaldehyde subsequently to initial flow cytometry and stained for Alexa488-anti-GFP prior to a second FACS. Antibodies used are listed in Supplemental Table 1 and FACS data were analyzed in Flowjo after flow cytometry on a BD Fortessa.

### Statistics

Means ± SEM for the number of observations are given where one mouse is one n. The number of experiments, *i.e*. occasions at which a number of mice (of both genotypes) were analysed, are also given. Comparisons of chance differences were done by Students’ t-test when compared with corresponding control or by ANOVA followed by Sidak’s or Dunnett’s multiple comparisons test as indicated using Prism 8 software.

## Supplementary information

Supplementary figures 1-10 and supplementary table 1
