# Supplementary material for: The Cdh5-CreERT2 transgene causes conditional Shb gene deletion in hematopoietic cells with consequences for immune cell responses to tumors
Source: Sci Rep. 2019 May 17;9:7548. doi: 10.1038/s41598-019-44039-z (PMC6525206; doi:10.1038/s41598-019-44039-z)
Supplement: Supplementary file 1 — Supplementary figures 1-10 and supplementary table 1 [file 41598_2019_44039_MOESM1_ESM.pdf]

Supplementary figures 1-10 and supplementary table 1.

The *Cdh5-CreERT2* transgene causes conditional *Shb* gene deletion in hematopoietic cells with consequences for immune cell responses to tumors.

Qi He[1], Xiujuan Li[1,2], Kailash Singh[1], Zhengkang Luo[1], Mariela Meija-Cordova[1], Maria Jamalpour[1], Björn Lindahl[1], Vitezslav Kriz[3], Reetta Vuolteenaho[4], Maria Ulvmar[5] and Michael Welsh[1]

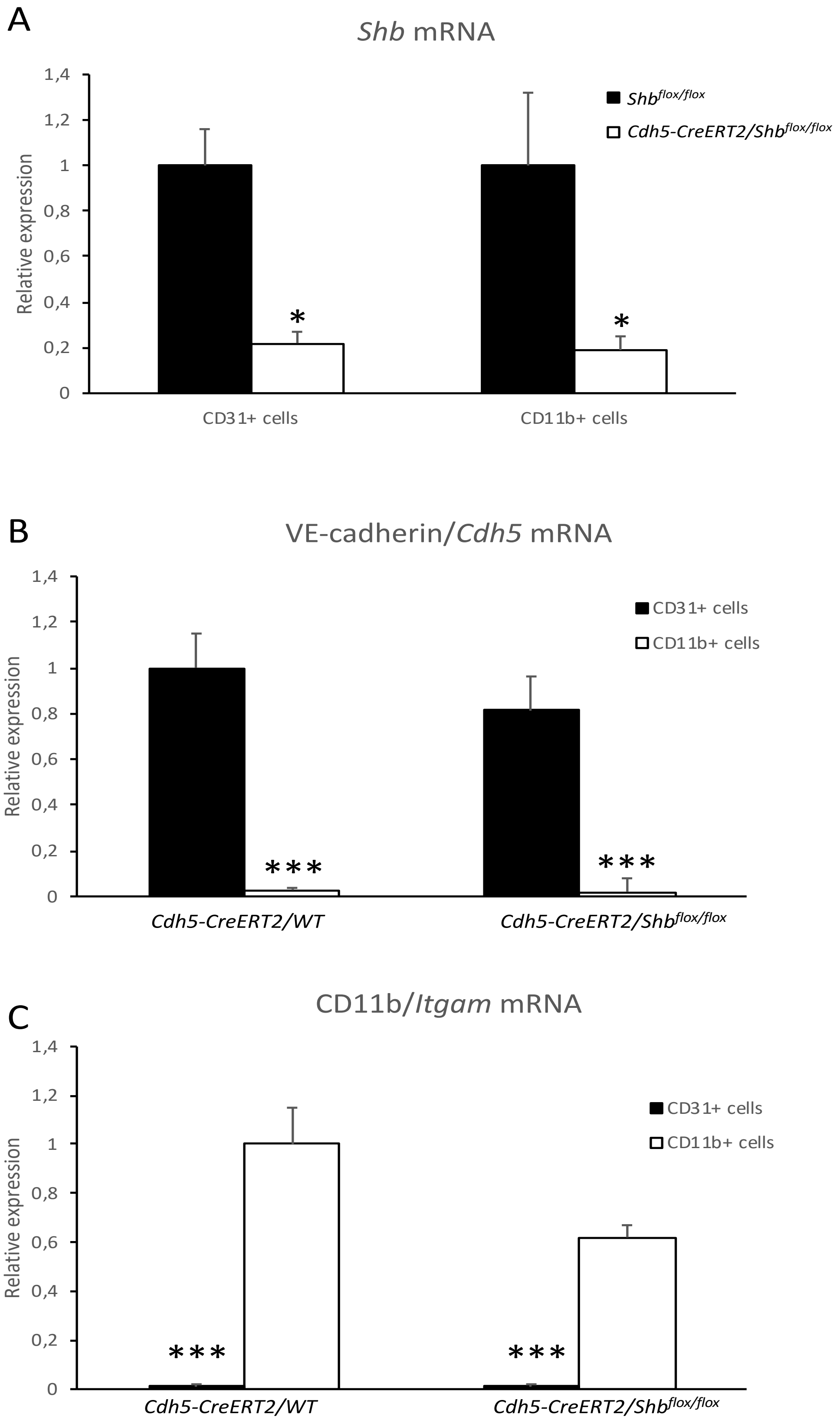

Supplementary Figure 1: *Shb*, VE-cadherin/*Cdh5* and CD11b/*Itgam* mRNA in isolated tumor CD31+ and CD11b+ cells from the indicated genotypes. Values are relative expression as means ± SEM. In A) *Shb*$^{flox/flox}$ *Shb* mRNA (after normalization for beta-actin) was set to 1 for the two cell types. N=3 for *Shb*$^{flox/flox}$ and N=10 for *Cdh5-CreERT2/Shb*$^{flox/flox}$. VE-cadherin/*Cdh5* mRNA (B) and CD11b/*Itgam* mRNA (C) for wild type (WT) and *Cdh5-CreERT2/Shb*$^{flox/flox}$ CD31+ and CD11b+ cells. Values are relative WT CD31+ and CD11b+ cells,

respectively. N=10. In A, * indicates $p < 0.05$ when compared with their respective control values by ANOVA and Dunnett's multiple comparisons test. In B, C, *** indicates $p < 0.001$ using a two-way ANOVA followed by Sidak's test for multiple comparisons.

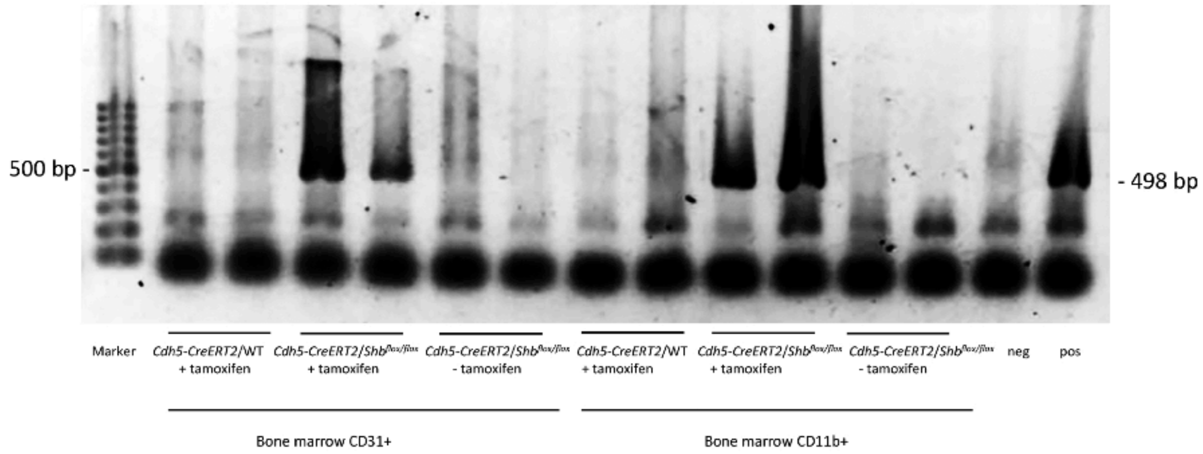

Supplementary Figure 2: Genotyping by PCR demonstrating floxing of the *Shb* gene in bone marrow CD31+ and CD11b+ cells isolated 4 days after the last tamoxifen injection. Primers were selectively chosen to demonstrate floxing and these will not amplify wild type or non-floxed DNA. The floxed 498 bp band is shown in the positive control next to the negative blank. *Cdh5-CreERT2*/WT and *Cdh5-CreERT2*/*Shb*$^{flox/flox}$ mice were treated with tamoxifen. Non-tamoxifen treated *Cdh5-CreERT2*/*Shb*$^{flox/flox}$ mice are also shown. Each lane represents an individual mouse. Molecular weight standard is shown to the left. The uncropped image is shown in Supplementary Figure 7.

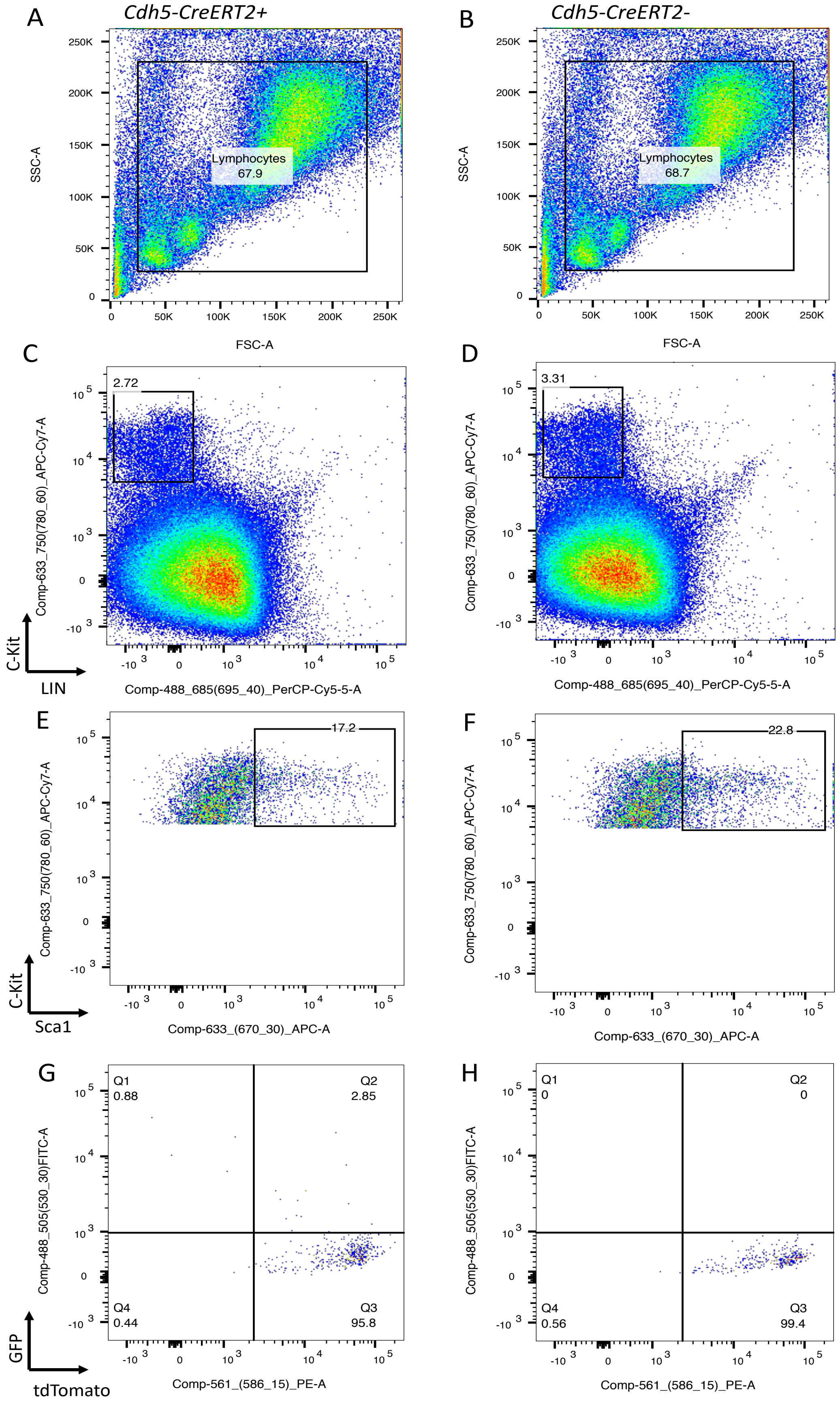

Supplementary Figure 3: FACS profiles and gating showing GFP+ K+S+L- cells. A,B) Total bone marrow populations. C,D) cKit+/Lin- cells. E,F) cKit+/Sca1+ from cKit+/Lin- cells. G,H) GFP/tdTomato cells from cKit+/Sca1+/Lin- cells. A,C,E,G show *Cdh5-CreERT2+* cells and B,D,F,H show *Cdh5-CreERT2-* cells.

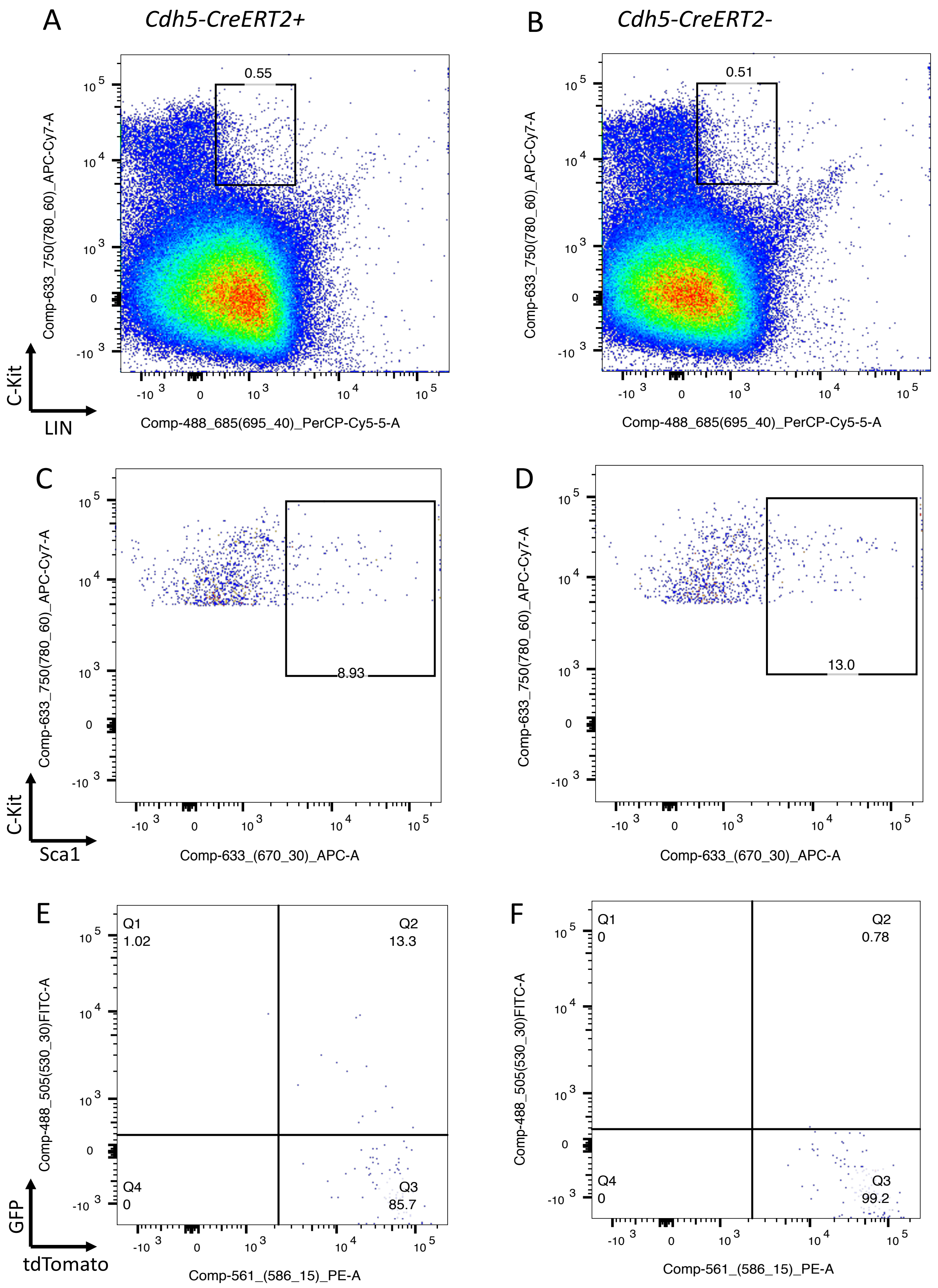

Supplementary Figure 4: FACS profiles and gating showing GFP+ cKit+, Sca1+, lineage+ bone marrow cells. A,B) cKit+/Lin- cells. C,D) cKit+/Sca1+ from cKit+/Lin- cells. E,F) GFP/tdTomato cells from cKit+/Sca1+/Lin- cells. A,C,E, show *Cdh5-CreERT2+* cells and B,D,F, show *Cdh5-CreERT2-* cells.

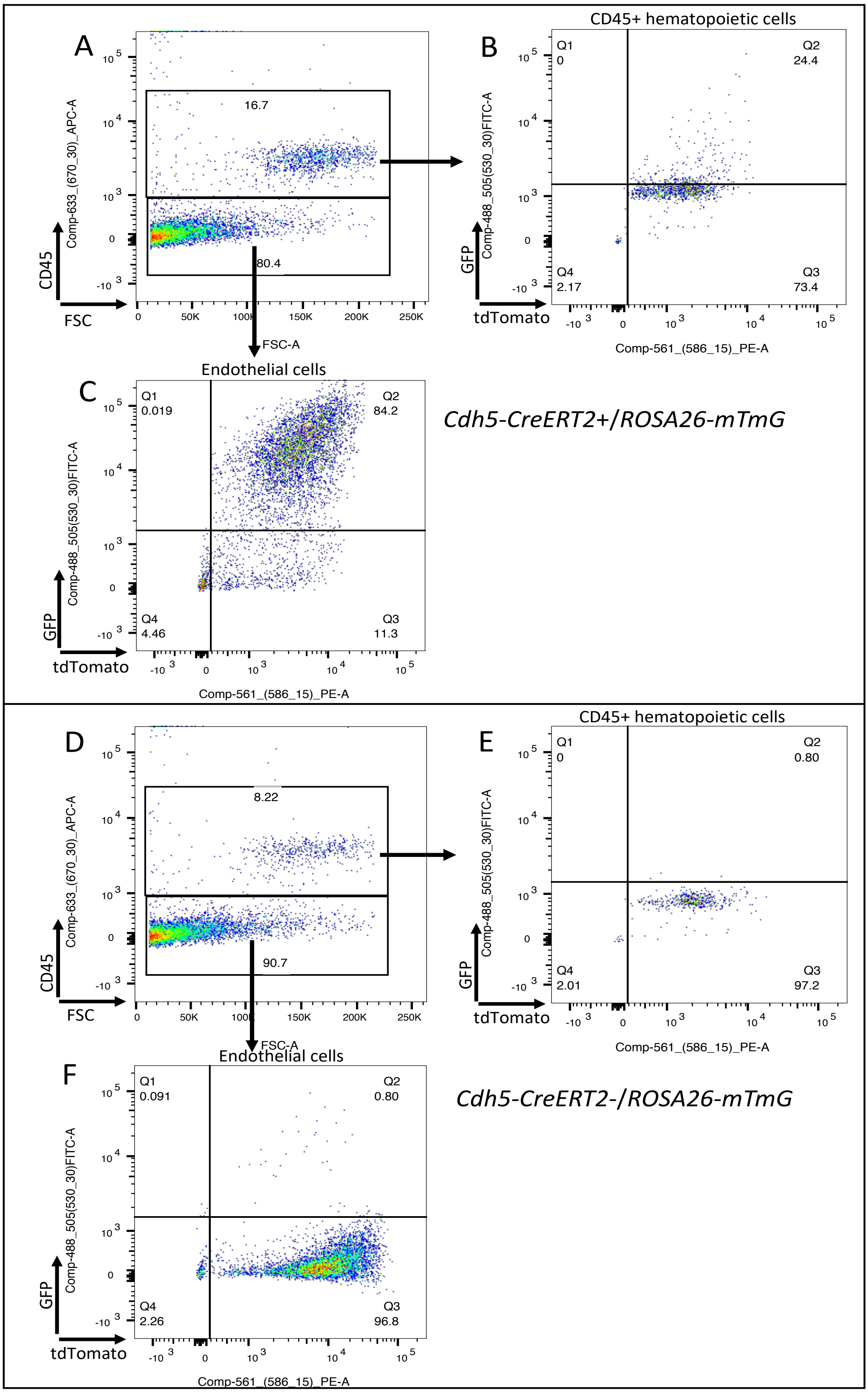

Supplementary Figure 5: FACS profiles and gating showing *Cdh5-CreERT2+/ROSA26-mTmG* (A-C) and *Cdh5-CreERT2-/ROSA26-mTmG* (D-F) mouse lung hematopoietic and endothelial cells. CD31 positive cells isolated from lung were fixed with paraformaldehyde and stained for CD45 (A,D). GFP and tdTomato in lung hematopoietic cells are shown in the two right panels (B,E) where non-CD45 positive cells (endothelial cells) are shown in the two lower panels (C,F). Note the much stronger tdTomato fluorescence in the endothelial cells compared with the hematopoietic cells when analysed under identical conditions in the same FACS run. Also note the difference in GFP positivity between the *Cdh5-CreERT2+* and *Cdh5-CreERT2-* hematopoietic cells.

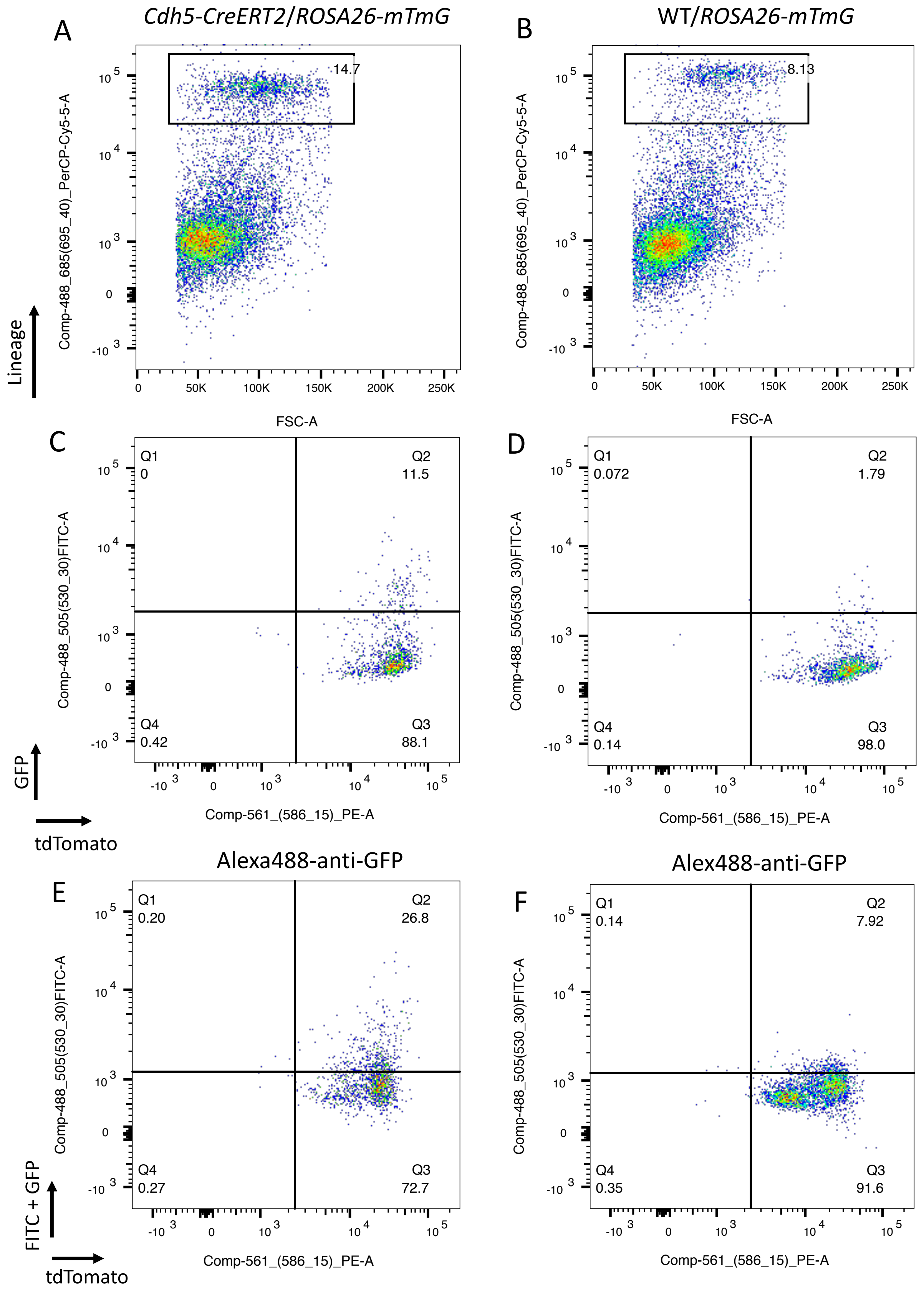

Supplementary Figure 6: FACS profiles and gating showing GFP+ spleen myeloid cells with or without Alexa488-anti-GFP staining. A,C,E *Cdh5-CreERT2+* and B,D,F *Cdh5-CreERT2-*. A,D show myeloid cells (Gr1, CD11b positive) and C,D GFP+ without or E,F with anti-GFP staining.

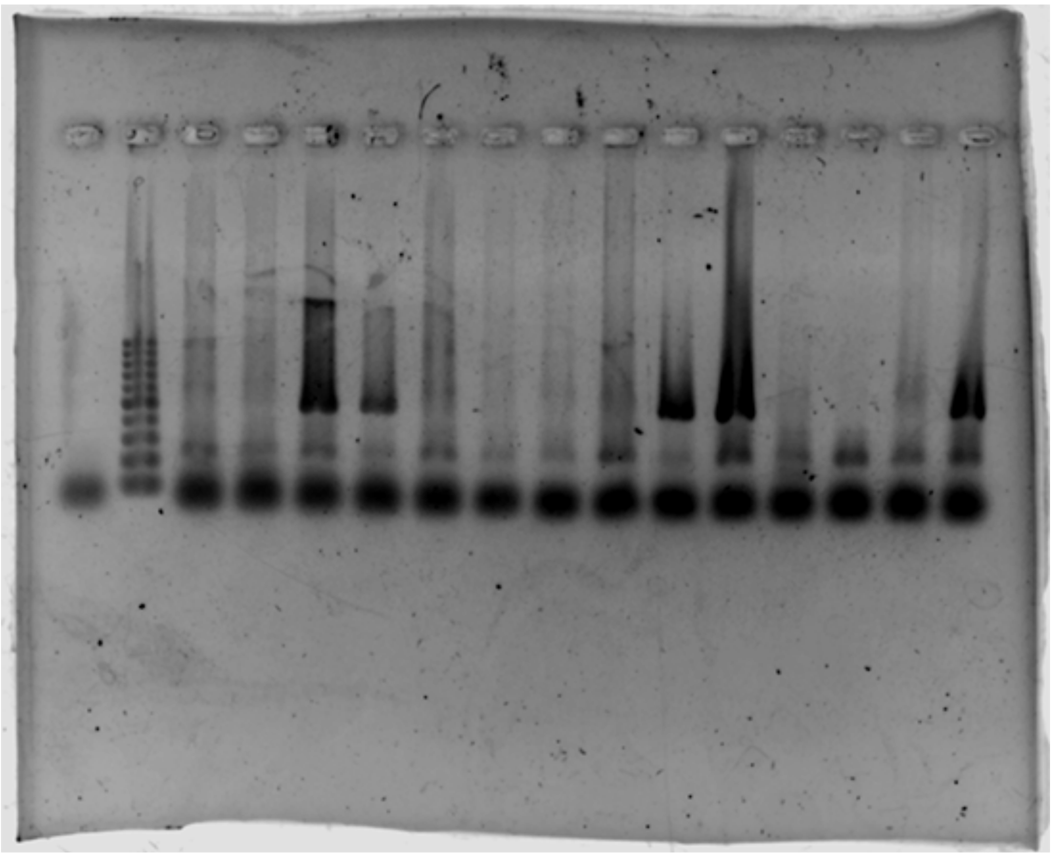

Supplementary Figure 7: Uncropped original version of Figure 3.

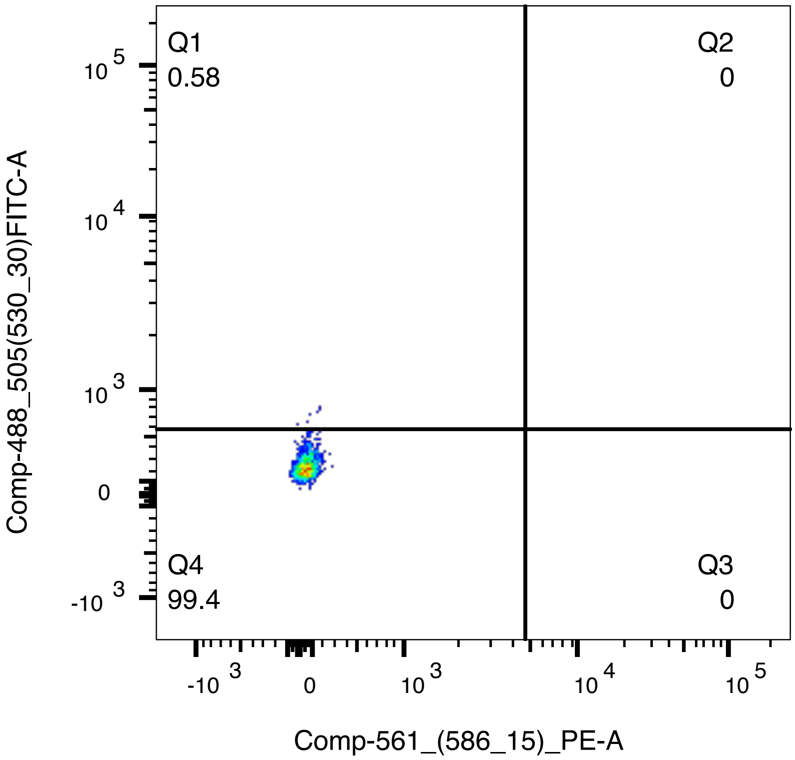

Supplementary Figure 8: Wild type mouse lacking *ROSA26-mTmG* and *Cdh5-CreERT2* showing GFP/tdTomato signals in K+S+L- bone marrow cells corresponding to Figure 3C. The FITC channel shows some signal indicating autofluorescence whereas the tdTomato signal is very weak.

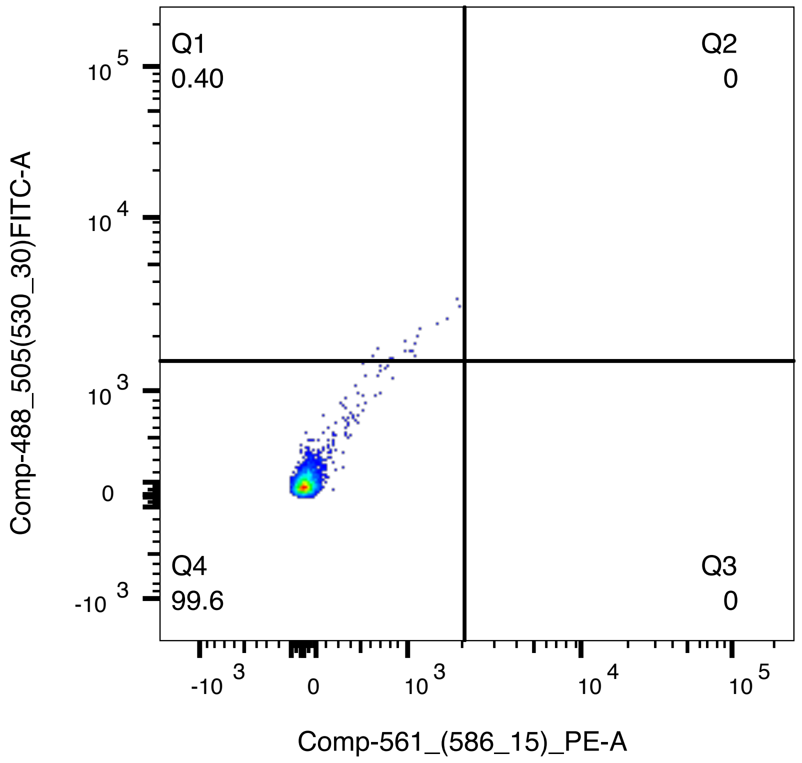

Supplementary Figure 9: Wild type mouse lacking *ROSA26-mTmG* and *Cdh5-CreERT2* showing GFP/tdTomato signals in the K+S+L+ cells corresponding to Figure 4C.

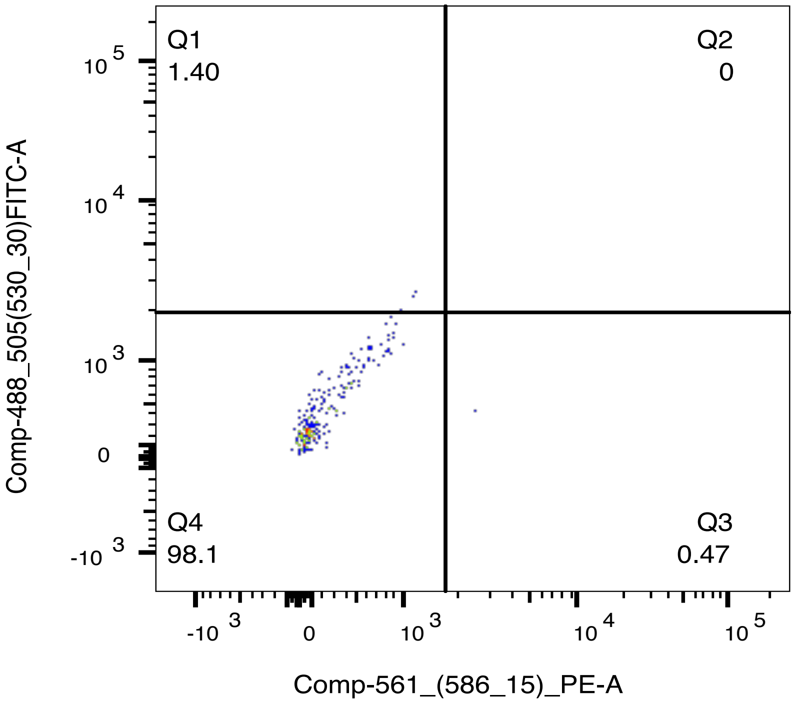

Supplementary Figure 10: Wild type mouse lacking *ROSA26-mTmG* and *Cdh5-CreERT2* showing GFP/tdTomato signals in spleen cells corresponding to Figure 3E.

Supplementary Table 1: Antibodies used.

Antibody	Product code	Supplier	Fluorophore
CD3	100243	Biolegend	
CD4	100401	Biolegend	
CD4	100405	Biolegend	FITC
CD8	100701	Biolegend	
Gr1	RM3000	Life Technologies	
CD11b	101201	Biolegend	
B220	16-0452-85	eBioscience	
CD19	115502	Biolegend	
Ter119	116201	Biolegend	
anti-rat secondary	405424	Biolegend	Percp-Cy5.5
c-Kit	47-1171-82	eBioscience	Apc-Cy7
Sca1	17-5981-81	eBioscience.	APC
FoxP3	4344419	Invitrogen	PE-Cy7
Anti-GFP	338008	Biolegend	Alexa488
CD45.2	558702	BD Pharmingen	APC
